# Whole genome sequence of *Tsukamurella tyrosinosolvens* extracted from metagenome of human pleural fluid enriched in Mycobacteria Growth Indicator Tube

**DOI:** 10.1128/mra.01518-25

**Published:** 2026-04-30

**Authors:** Rameez Moidu Jameela, Mayur Mahindra Kedare, Rehbar Khan, Nehal Dhankad, Rishav Kumar Sinha, Amrutraj Zade, Sanchi Shah, Anirvan Chatterjee

**Affiliations:** 1 HaystackAnalytics Private Limited 729199 , Navi Mumbai, India; 2 Department of Respiratory Medicine, Netaji Subhash Chandra Bose Subharti Medical College 78201, Meerut, India; Fluxus Inc., Sunnyvale, California, USA

**Keywords:** *Tsukamurella tyrosinosolvens*, metagenome assembled genome, pleuritis, misdiagnosis of *Mycobacterium tuberculosis*, metagenomic binning, emerging pathogen, Mycobacteria Growth Indicator Tube

## Abstract

Misdiagnosis of emerging pathogen *Tsukamurella tyrosinosolvens* is common due to phenotypic similarity with *Mycobacterium tuberculosis* (MTB). We report a high-quality, near-complete genome of *T. tyrosinosolvens* from pleural fluid enriched in Mycobacteria Growth Indicator Tube. The genome of this clinically successful strain can be studied to understand pathogenesis and diagnostic challenges.

## ANNOUNCEMENT

Tuberculous pleuritis is a common extrapulmonary manifestation often presented with nonspecific symptoms (1, 2). Misidentification of *Tsukamurella* as *Mycobacterium tuberculosis* (MTB) is a well-recognized diagnostic challenge because the two genera share several morphological, biochemical, and clinical features (3–5). From the left-over data of a commercial whole-genome sequencing-based test on a MTB-suspected pleural fluid, we obtained a near-complete genome of *Tsukamurella tyrosinosolvens*. This genome can be explored for further studies on pathogenic features of this species.

**Fig 1:**
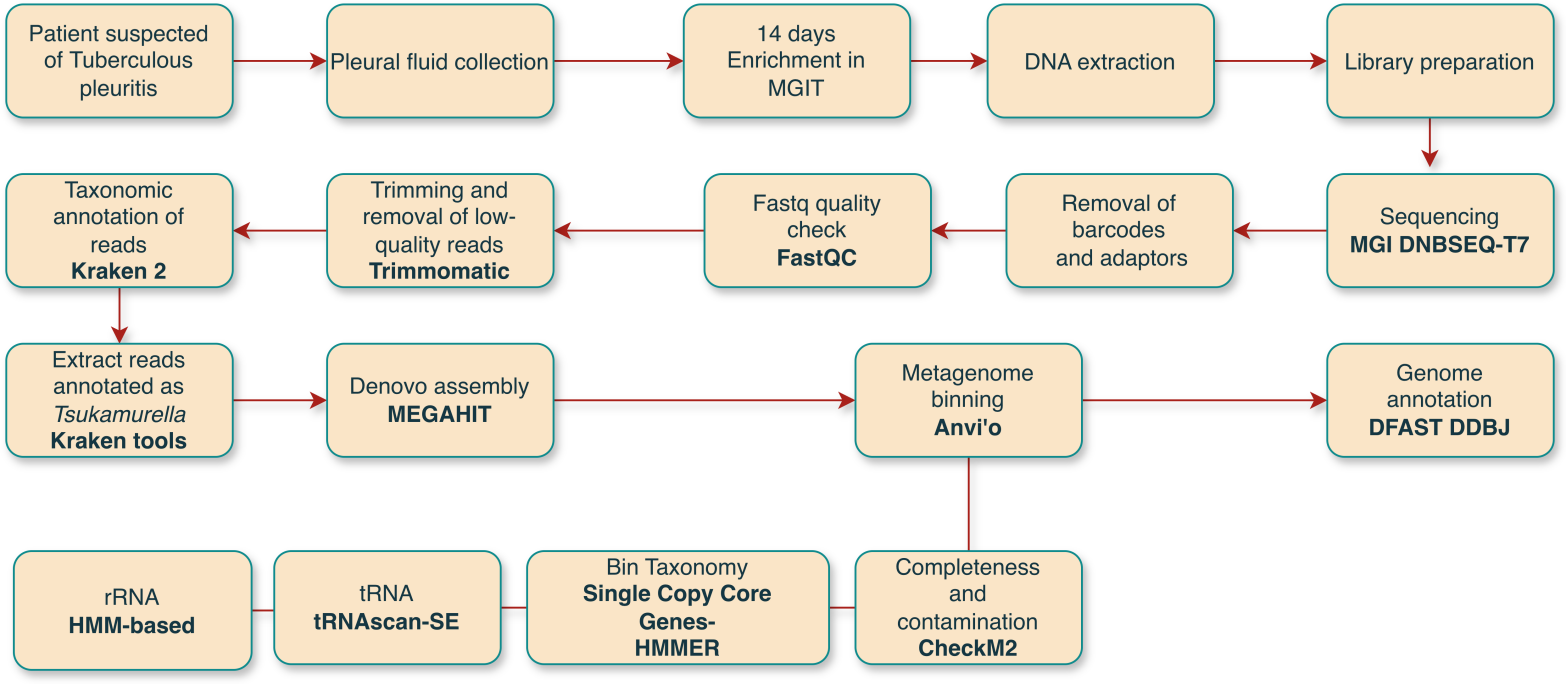
Details of sample processing and analyses employed. Software/tools used are in bold text. Direction of arrows connecting the text boxes is consistent with the directional flow of processing. Lines without arrows connect multiple steps happening within a single software.

Pleural fluid was obtained from the patient and collected in a sterile container on 11 August 2025 at Meerut, Delhi, India. The sample was inoculated in a Mycobacteria Growth Indicator Tube (MGIT) (6) to enrich Mycobacteria that may present in the sample. On day 15, total DNA was extracted using Qiagen DNeasy UltraClean Microbial Kit (Cat. no. 12224, Qiagen, Germany) with modifications as suggested elsewhere (7). The sequencing library was prepared from this extracted DNA using the Nextera XT DNA Library Preparation Kit (Illumina Cat. No. FC-131-1096) and sequenced using DNBSEQ-T7 machine from MGI as per manufacturer’s protocol. Quality of such generated paired-end reads was checked using FastQC 0.12.1 (8). Low-quality reads and adaptors were removed using Trimmomatic 0.39 (9) (ILLUMINACLIP:TruSeq3-PE.fa:2:30:10:2:True LEADING:3 TRAILING:3 MINLEN:36). The trimmed reads annotated as *Tsukamurella* by Kraken 2 2.1.3 (10) were extracted and assembled using MEGAHIT 1.2.9 (11) (--min-contig-len 500); 76 contigs generated were subjected to metagenomic binning using interactive interface from Anvi’o 8 (12). The taxonomy of the bin generated was checked through HMM search of single copy core genes from Anvi’o SCG taxonomy database (version 214.1), as well as based on the 16S rRNA gene sequence detected. Completeness and contamination of the bin was assessed using CheckM2 1.1.0 (13). The genome was annotated using DFAST 1.3.6 (14). All tools used with default parameters unless mentioned otherwise. Steps involved in the process are summarized in Fig. 1.

Details of reads, contigs, and bins generated in the process are provided in Table 1. This bin was annotated as *Tsukamurella tyrosinosolvens*. CheckM2 inferred a completeness of 100% and contamination of 1.92% for this bin. Based on the total of 4.665 coding sequences, CheckM2 inferred a coding density of 0.931. This high-quality metagenome assembled genome (MAG) with 1000X coverage and N50 value of 196,439 bp has a total sequence length of 4,751,425 bp with 72% GC content, as expected for this organism. 5S, 16S, and 23S rRNA genes were detected along with genes for tRNA that code 20 amino acids. The MAG is named *T. tyrosinosolvens* HAPL-NEOL2MGTWRFYC902. Since the specimen was obtained during standard procedure for diagnosis, ethical committee approval was not needed; hence, IRB review was not conducted for this manuscript.

**TABLE 1:** Quantitative overview of the process of MAG generation and quality check

Reads	Number of reads	Percentage (%)
Total reads	54,446,796	100
Trimmed reads	54,407,708	99.93
Reads annotated as *T. tyrosinosolvens^a^*	44,946,484	82.55

Assembly, binning and QC	Value	Percentage (%)
Total contigs generated	76	100
Number of bins obtained	1	NA^*c*^
Number of contigs in the bin	50	65
Number of contigs from bin annotated as *T. tyrosinosolvens^b^*	50	100
Total consensus length of the bin	4,751,425 bp	NA
N50	196,439 bp	NA
Length of the largest contig	754,573 bp	NA
CheckM2		
Completeness	NA	100
Contamination	NA	1.92

^*a*^ Reads annotated using Kraken 2.

^*b*^ Contigs annotated within Anvi’o through HMM search of single copy core gene database.

^*c*^ NA, not applicable.

## Data Availability

The MAG sequence has been submitted to DDBJ (accession BAAJPF010000001–BAAJPF010000050), linked to biosample SAMD01793676 under the bioproject PRJDB40014. The raw sequencing data is available at the DRA with accession DRR895690.
